# Effect of Silver Powder Microstructure on the Performance of Silver Powder and Front-Side Solar Silver Paste

**DOI:** 10.3390/ma17020445

**Published:** 2024-01-17

**Authors:** Xianglei Yu, Hu Sun, Zhuo Qian, Weichao Li, Wei Li, Fuchun Huang, Junpeng Li, Guoyou Gan

**Affiliations:** 1 Faculty of Material Science and Engineering, Kunming University of Science and Technology, Kunming 650093, China; 20181130006@stu.kust.edu.cn (X.Y.); 20212230133@stu.kust.edu.cn (H.S.); zhuoqian@kust.edu.cn (Z.Q.); 2 R&D Center of Yunnan Tin Group (Holding) Co., Ltd., Kunming 650093, China; weicli@126.com; 3 Sino-Platinum Metals Co., Ltd., Kunming 650093, China; li_gzu@126.com (W.L.); hfc@ipm.com.cn (F.H.)

**Keywords:** silver powder, solar cell, aggregation growth, crystal growth, sintering activity

## Abstract

Silver powder, as the primary component of solar silver paste, significantly influences various aspects of the paste’s performance, including printing, sintering, and conductivity. This study reveals that, beyond the shape and size of the silver powders, their microstructure is a critical factor influencing the performance of both silver powders and silver pastes in solar cell applications. The growth process leads to the formation of either polycrystalline aggregated silver powder or crystal growth silver powder. Analyzing the performance characteristics of these different microstructures provides guidance for selecting silver powders for silver pastes at different sintering temperatures. Polycrystalline aggregated silver powder exhibits higher sintering activity, with a sintering initiation temperature around 450 °C. The resulting silver paste, sintered at 750 °C, demonstrates a low sheet resistance of 2.92 mΩ/sq and high adhesion of 2.13 N. This silver powder is suitable for formulating silver pastes with lower sintering temperatures. The solar cell electrode grid lines have a high aspect ratio of 0.37, showing poor uniformity. However, due to the high sintering activity of the silver powder, the glass layer dissolves and deposits more silver, resulting in excellent conductivity, a low contact resistance of the silver electrode, a low series resistance of the solar cell of 1.23 mΩ, and a high photoelectric conversion efficiency of 23.16%. Crystal growth silver powder exhibits the highest tap density of 5.52 g/cm^3^. The corresponding silver paste shows improved densification upon sintering, especially at 840 °C, yielding a sheet resistance of 2.56 mΩ/sq and adhesion of 3.05 N. This silver powder is suitable for formulating silver pastes with higher sintering temperatures. The solar cell electrode grid lines are uniform with the highest aspect ratio of 0.40, resulting in a smaller shading area, a high fill factor of 81.59%, and a slightly higher photoelectric conversion efficiency of 23.17% compared to the polycrystalline aggregated silver powder.

## 1. Introduction

Photovoltaic power generation, as a method to harness abundant, clean, and reliable renewable energy, has seen rapid development against the backdrop of increasing global energy demand [1,2]. In 2022, the global photovoltaic installed capacity reached 230 GW, marking a substantial year-on-year growth of 35.3%. Within this landscape, crystalline silicon solar cells hold a dominant share, exceeding 90%, owing to their advantages such as low cost per unit of electricity, mature production processes, and extended lifespan [3,4,5,6,7,8,9,10,11]. The silver paste, especially the front-side silver paste, plays a pivotal role as a key material for fabricating electrodes in crystalline silicon solar cells. Its performance directly influences various aspects of solar cells, including series resistance (R_s_), short-circuit current (I_sc_), fill factor (FF), and photoelectric conversion efficiency (Eta) [12,13,14,15,16,17]. Typically, front-side silver paste comprises organic carriers, glass powder, and silver powder. The organic carrier primarily modulates the rheological properties of the silver paste to meet the dimensional and integrity requirements of fine grid lines for screen printing [18,19,20,21,22,23,24]. The glass powder serves to etch the SiN_x_ antireflection layer and establish a favorable interface contact with the cell emitter [25,26,27,28,29,30,31,32,33,34]. Silver powder, constituting over 85% of the mass of solar silver paste, is the conductive phase and major component. The morphology, size, and sintering performance of silver powder directly impact the particle stacking before sintering, the formation and densification of the silver electrode conductive path during sintering, and the post-sintering properties of the electrode grid lines, including the appearance, internal structure, resistivity, and contact resistance [35,36,37,38,39,40]. These aspects make silver powder a crucial factor in the study of the sintering process of solar silver paste.

The emitter of a solar cell employs high concentrations of ion doping to enhance photoelectric conversion efficiency. To mitigate the impact of high-temperature sintering on solar cell performance, the metallization sintering process for solar silver paste necessitates the use of as low a sintering temperature and as short a sintering time as possible. Typically, a belt furnace is employed for sintering, with the peak temperature ranging between 750 and 850 °C, which is below the melting point of metallic silver. The peak sintering time is measured in seconds [41,42]. During the sintering of the front-side solar silver paste, two main processes occur. Firstly, the silver particles undergo mutual sintering, forming conductive paths and densification [43,44,45]. Secondly, the glass powder softens, melts, and flows onto the silicon layer, establishing the interface connection between the silver electrode and the silicon solar cell [46,47,48,49]. The former process determines the resistivity of the silver electrode post-sintering, requiring the close packing of silver particles and high sintering activity. The latter process influences the adhesion and contact resistance of the silver electrode post-sintering, requiring that the sintering densification of the silver particles occurs later than the softening and flow of the glass powder. This prevents the reduction in pores after silver particle sintering, which could lead to glass residue in the silver layer [50,51,52,53,54]. This not only increases the resistivity of the silver electrode but also results in an insufficient glass interlayer at the silver–silicon interface [55,56,57,58,59]. Hence, it is generally considered that silver powder suitable for front-side solar silver paste should possess an average particle size of 1–1.5 μm, a narrow size distribution, and high sphericity.

Current research on silver powder focuses on enhancing the sintering performance of silver paste through different shapes, particle sizes, and combinations of silver powders, the addition of submicron silver powders, and the construction of nanostructures [60,61]. These efforts aim to achieve denser silver electrodes and higher aspect ratios of silver grid lines [62,63,64]. Libin Mo et al. [24], investigated the effects of incorporating 0–10 wt% submicron spherical silver powder (average particle size of 0.3 μm) into a micrometer-sized spherical silver powder (average particle size of 1 μm) on the rheological properties of silver paste, printed grid dimensions, sintering behavior, and the cell’s electrical performance. The results indicated that the addition of submicron silver powder can improve the sintering performance of silver powder, reduce the porosity of the silver electrode surface, lower bulk resistivity, and increase the size of the silver microcrystals precipitated on the silicon surface, thereby reducing contact resistivity. However, the excessive addition of submicron silver powder can adversely affect the printability of the silver paste, leading to a decrease in the aspect ratio of the printed grid lines, an increase in series resistance, and a decrease in photoelectric conversion efficiency. Therefore, the addition of an appropriate amount of submicron silver powder needs to be considered holistically. Yongsheng Li et al. [65] developed microcrystalline spherical silver particles (SP-a) with internal pores. The study demonstrated that SP-a exhibits excellent sintering activity, forming a dense sintered body. At the Ag–Si interface, silver nanoparticles are formed, improving the silver–silicon contact. Photovoltaic cells constructed using SP-a exhibit low series resistance and high photoelectric conversion efficiency (19.26%).

The prior investigations on the performance of silver powders, particularly those describing micrometer-sized spherical silver powders, predominantly focused on their shape and particle size [66,67]. However, a comprehensive exploration of the impact of the silver powder’s crystal structure on performance is lacking, leading to a relatively superficial differentiation of silver powders. With the advancement of solar cell technology and the gradual approach of photovoltaic conversion efficiency in crystalline silicon solar cells to theoretical limits, there is an increasing demand for more nuanced properties in the silver paste. For instance, to mitigate the influence of thermal processing on the doping concentration of the cell emitter during solar cell fabrication, there is a growing need for silver pastes with lower sintering temperatures and shorter sintering times [68]. Moreover, to reduce the shading area of the silver electrode grid lines and enhance the active area for power generation, the grid line printing width was reduced to 15 μm. To ensure the conductivity of these grid lines, it is imperative for the silver paste to exhibit a higher aspect ratio after sintering, while controlling the sintering shrinkage to prevent grid line breakage. This paper delves into the influence of the crystal structure of the silver powder on sintering activity, specific surface area, tap density, the sintering performance of the silver paste, the aspect ratio of the electrode grid lines, and solar cell performance. By elucidating the correlation between silver powder microstructure and performance, this research refines the categorization of silver powders, providing insights for the formulation of silver pastes with more nuanced properties. This work aims to contribute to the development of silver pastes tailored to meet the increasingly detailed requirements of solar cell technologies.

## 2. Materials and Methods

### 2.1. Materials

The silver powder used in this study was custom made by Sino-Platinum Metals Co., Ltd. (Kunming, China), featuring the various microstructures of micron-sized spherical silver powder. The glass powder employed was prepared using the melt-quenching method, with raw materials sourced externally without purification processing, comprising TeO_2_ (≥99%, Aladdin Reagent (Shanghai) Co., Ltd., Shanghai, China), Bi_2_O_3_ (≥99.0%, Aladdin Reagent (Shanghai) Co., Ltd., Shanghai, China), B_2_O_3_ (≥98%, Aladdin Reagent (Shanghai) Co., Ltd., Shanghai, China), ZnO (≥99%, Aladdin Reagent (Shanghai) Co., Ltd., Shanghai, China), Al_2_O_3_ (≥99%, Aladdin Reagent (Shanghai) Co., Ltd., Shanghai, China). The materials were proportionally weighed in a mass ratio of TeO_2_:Bi_2_O_3_:B_2_O_3_:ZnO:Al_2_O_3_ = 15:50:20:10:5, mixed thoroughly in an agate mortar, and melted at 1200 °C for 30 min. The molten glass was quenched in deionized water at room temperature, and the obtained glass chunks were ball-milled and sieved to yield glass powder with an average particle size of 2.4 μm. The organic vehicle used consisted of a blend of ethyl cellulose (CP, Aladdin Reagent (Shanghai) Co., Ltd., Shanghai, China), terpineol (≥95%, Aladdin Reagent (Shanghai) Co., Ltd., Shanghai, China), butyl carbitol (≥99%, Shanghai Wokai Chemical Reagent Co., Ltd., Shanghai, China), butyl phthalate (≥99.5%, Sinopharm Chemical Reagent Co., Ltd., Beijing, China), silane coupling agent KH-570 (≥98%, Sinopharm Chemical Reagent Co., Ltd., Beijing, China), and hydrogenated castor oil (Aladdin Reagent (Shanghai) Co., Ltd., Shanghai, China). The silver paste was formulated with a mass ratio of silver powder, glass powder and organic vehicle of 43:1:6, initially weighed and mixed, followed by further refinement through a three-roll mill to achieve a fineness of <5 μm.

### 2.2. Printing and Metallization of Ag Pastes

Various test patterns were printed on P-type monocrystalline silicon solar cells with a surface SiN_x_ antireflection coating using a screen-printing machine. The size of solar cell was 182 × 182 mm, with an 80 nm thick SiN_x_ antireflection layer possessing a refractive index ranging from 2.0 to 2.35. The sheet resistance of the cell emitter was 80 Ω/sq, and the emitter depth was less than 0.2 μm. For sheet resistance testing, 10 parallel lines, each 2 mm wide and 100 mm long, were printed. Adhesion testing involved printing square patterns of 20 × 20 mm, while performance testing of the solar cells utilized printed patterns consisting of 9 main grid lines and 104 secondary grid lines with a width of 15 μm. Following printing, the solar cells were allowed to stand at room temperature for 10 min to allow the silver paste to level adequately. Subsequently, the cells were dried at 150 °C for 20 min and then subjected to metallization of the front silver paste through sintering in a continuous belt furnace. The peak sintering temperature was denoted as T_0_, and the heating temperatures in different zones of the belt furnace were as follows: 300 °C/300 °C/300 °C/500 °C/590 °C/600 °C/T_0_-100 °C/T_0_/T_0_-15 °C.

### 2.3. Measurement and Characterization

The microstructures of the silver powder, as well as the surface and cross-section of the silver electrode, were observed using field emission scanning electron microscopy (FESEM, Nova Nano SEM 450, FEI, Hillsboro, OR, USA). The particle size distribution of the silver powder was determined using image software (Image J, 1.54d). The number of measured silver particles exceeded or equaled 200. The specific surface area of the silver powders was analyzed using a surface area porosity analyzer (BET, BSD-PS1/2/4, BSD INSTRUMENT, Beijing, China). The crystal structure of the silver powder was identified through X-ray diffraction (XRD, Empyrean, PANalytical B.V., Almelo, The Netherlands). Thermal properties were assessed using differential scanning calorimetry (DSC, Q2000, TA Corporation, New Castle, DE, USA) in air with a heating rate of 10 °C/min. The square resistance of the silver grids was measured with a multifunction digital four-probe tester (ST-2258C, Suzhou Lattice Electronics Co., Ltd., Suzhou, China). Prior to conducting the adhesion test on the silver paste, a 1 mm wide surface-tinned copper strip (photovoltaic welding strip) was welded onto the prepared 20 × 20 mm square silver layer. The welding process occurred at 350 °C, and prior to welding, the application of flux was necessary. Following the welding procedure, the strip underwent a 180° bend, and the maximum force required for delamination of the silver layer was measured along the horizontal direction using a 10N capacity digital push–pull force gauge (ZDF-10, Shanghai Shangcen Precision Instrument Co., Ltd., Shanghai, China). The aspect ratio of the silver grids was determined using a 3D digital microscope (Zeta-20, Zeta Instruments, Inc., San Jose, CA, USA).

## 3. Results and Discussion

### 3.1. Microstructure and Performance Analysis of Silver Powder

#### 3.1.1. Microstructure of Silver Powder

**Figure 1:**
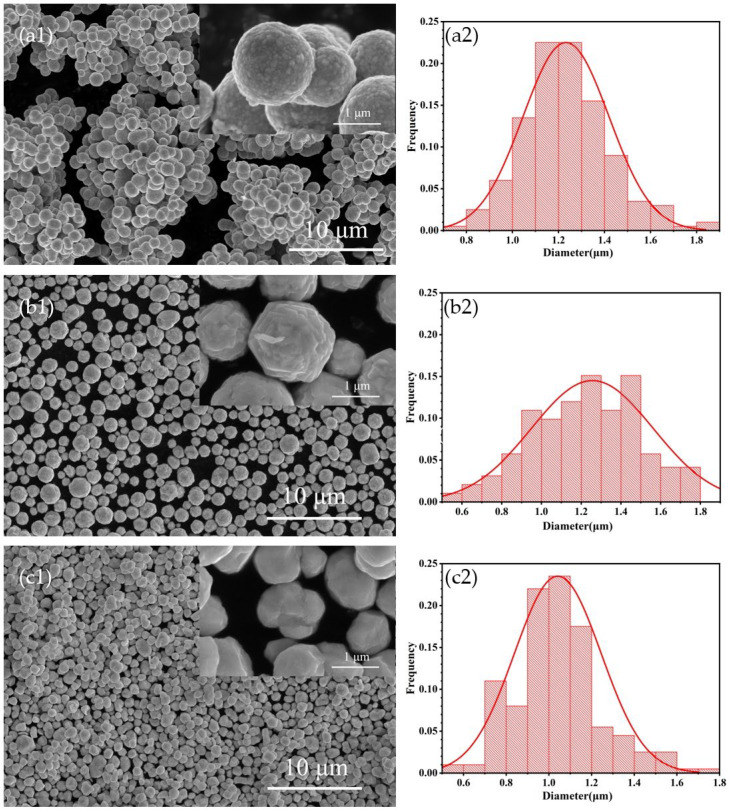
SEM images of silver powders (**a1**) S1, (**b1**) S2, (**c1**) S3; particle size distributions of silver powders (**a2**) S1, (**b2**) S2, (**c2**) S3.

This study selected three silver powders, denoted as S1, S2, and S3, with similar particle sizes and shapes but distinct crystal structures, and prepared by the liquid phase reduction method. The microstructures and particle sizes of the silver powders were observed using field emission scanning electron microscopy (FESEM). As shown in Figure 1(a1,c1), the surface of the S1 silver powder is characterized by the aggregation of numerous silver crystallites, each in the size range of several tens of nanometers. Despite a non-smooth surface, S1 exhibits high sphericity with no sharp edges. For the S2 silver powder, as depicted in Figure 1(a2,c2), larger silver crystallites aggregate on the surface, resulting in higher roughness and an almost spherical appearance with a few angular features. In the case of the S3 silver powder, the surface is predominantly formed through crystal growth, presenting a smooth yet polyhedral appearance with edges. The average particle sizes for S1, S2, and S3 are approximately 1.23 μm, 1.26 μm, and 1.04 μm, respectively. S1 and S3 exhibit a higher concentration in particle size distribution, with S1 displaying a distribution closest to a normal distribution. Conversely, the particle size distribution for S2 is less concentrated, with a more uniform distribution across different particle sizes.

**Figure 2:**
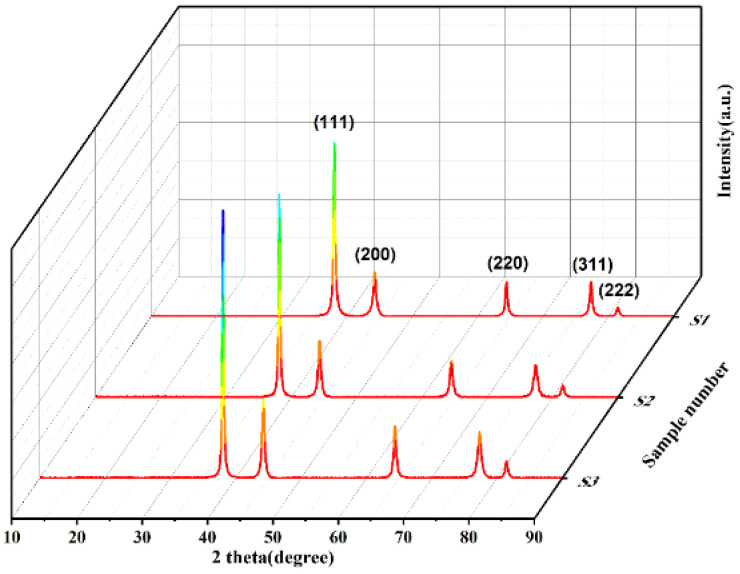
XRD diffraction patterns of spherical silver powders S1–S3.

The X-ray diffraction (XRD) patterns of silver powders S1 to S3 were examined, and the results are presented in Figure 2. It is evident that the diffraction peaks for all three silver powders correspond precisely to the standard PDF card for silver (PDF#87-0597), without any impurity peaks, indicating the purity of the silver in each powder. Additionally, an analysis of the three main crystallographic planes’ peak intensities and full-width at half-maximum (FWHM) values for silver powders S1 to S3, as summarized in Table 1, reveals notable changes in the strongest (111) crystallographic plane of S1, the peak intensity decreases, and the FWHM increases, indicative of broadening diffraction peaks. Typically, peak broadening occurs when the crystallite size is below 100 nm or when there are microstrains present in the sample. Since silver powders prepared via the liquid-phase reduction method generally do not undergo mechanical processing, microstrains are unlikely. This observation, coupled with the SEM magnified image in Figure 1(a1), suggests that silver powder S1 is composed of aggregated small grains, each smaller than 100 nm.

#### 3.1.2. Analysis of the Growth Process of Silver Powder

**Figure 3:**
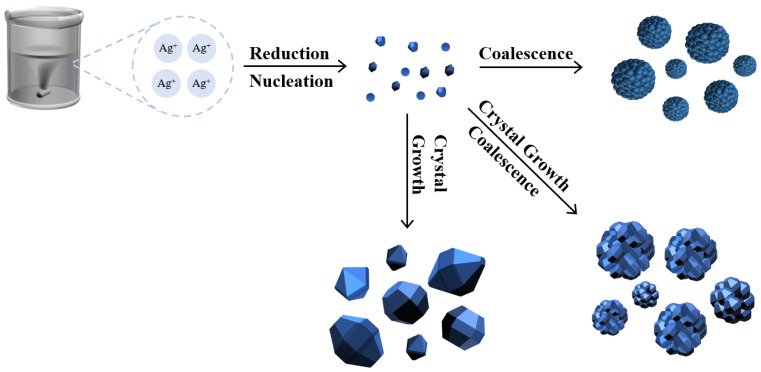
Schematic diagram of silver powder growth process.

The liquid-phase reduction method for silver powder involves two main steps: nucleation and growth. According to classical nucleation theory, crystalline nuclei begin to form in a solution when the solution’s supersaturation surpasses a critical threshold that overcomes the nucleation barrier [69]. Once nuclei are formed, the powder enters a growth stage, which occurs through two mechanisms. In one mechanism, small nanocrystalline nuclei in the solution aggregate directly, and due to the high surface energy at the contact points between particles, silver grows by bonding the particles together. This aggregation–bonding process repeats, leading to the continuous growth and aggregation of silver powder particles. The other mechanism involves direct growth along crystal faces. Given silver’s face-centered cubic crystal structure, Fm3m space group symmetry, and minimal variation in surface energies across different crystal faces, silver particles tend to grow into quasi-spherical polyhedra without growth-orientation intervention. The schematic diagram of the silver powder growth process is illustrated in Figure 3. Silver powder S1 represents a typical polycrystalline aggregation type, S3 reflects the crystal growth-type silver powder, and S2 is a hybrid type where both crystal growth and aggregation occur. In the preparation of silver powder, when the rate of crystal aggregation exceeds the rate of crystal growth, particularly in highly supersaturated reaction solutions leading to explosive nucleation in the early stages, the predominant mechanism is particle aggregation, resulting in silver powder resembling S1. Conversely, when the reaction solution has a lower supersaturation level, and the addition of the solution is prolonged, by limiting the initial nucleation or the aggregation process with the use of protective agents, crystal growth becomes the dominant mechanism, yielding a silver powder similar to S3. It is noteworthy that excessive protective agent content or exceptionally low solution supersaturation can lead to preferential crystal growth, resulting in non-spherical particles such as flakes, rods, or branched structures.

#### 3.1.3. Analysis of Macroscopic Physical Properties of Silver Powder

The macroscopic physical properties of silver powders S1–S3 are presented in Table 2. Tap density and specific surface area are two crucial performance parameters for solar silver pastes. Higher tap density indicates the silver powder’s ability to form denser packing, resulting in a more compact silver layer during paste sintering. A smaller specific surface area suggests a reduced surface area requiring wetting, leading to a lower organic vehicle content in rolled pastes with similar flow characteristics and fewer voids left after the organic vehicle volatilizes during sintering. Generally, for micrometer-sized silver powders with similar surface conditions, larger average particle size corresponds to higher tap density and smaller specific surface area. Although the average particle sizes of the silver powders S1 and S2 are close and both larger than those of silver powder S3, the measured tap density follows the order S3 > S2 > S1, while the specific surface area follows S3 < S2 < S1. This observation is speculated to be due to the presence of surface grains of different sizes on silver powders S1 and S2, contributing to an increased specific surface area. Silver powder S2, with some smooth surfaces, exhibits a smaller specific surface area than S1. Additionally, the existence of surface grains implies more grain boundaries on the surfaces of S1 and S2, indicating higher surface energy. Elevated surface energy tends to promote particle aggregation, resulting in larger interparticle gaps and reduced compaction during packing, leading to lower tapped density. Similarly shaped and sized silver powders exhibit significant macroscopic differences in performance. Therefore, in selecting silver powders, it is imperative to consider not only their shape and size but also their microstructure.

#### 3.1.4. Thermal Performance Analysis of Silver Powder

**Figure 4:**
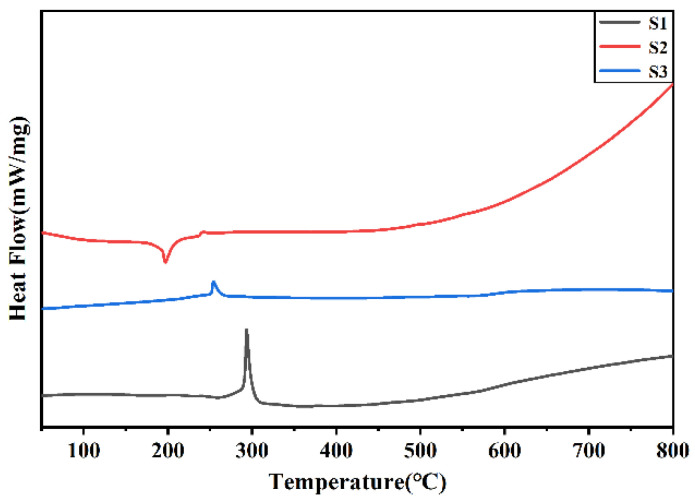
DSC curve of silver powders S1–S3.

The DSC test results for silver powders S1–S3 are illustrated in Figure 4. Combined with the TG curve shown in Supplementary Figure S1, silver powder S1 exhibits a prominent exothermic peak around 300 °C, accompanied by noticeable mass loss. Typically, mass loss in thermal behavior suggests either volatilization or decomposition. However, since volatilization is usually endothermic, it is speculated that a decomposition exothermic reaction has occurred. The most common decomposition exothermic reaction related to silver is the decomposition of Ag_2_O, as indicated in Equation (1). Due to the standard Gibbs free energy of formation of Ag_2_O becoming positive above 200 °C, silver undergoes spontaneous oxidation only at low temperatures, and above 250 °C, Ag_2_O decomposes back into silver, releasing heat. Silver powder S2 shows a small endothermic peak around 200 °C, accompanied by mass loss, suggesting the possible volatilization or decomposition of residual organic dispersants in S2. At around 250 °C, both silver powders S2 and S3 exhibit small exothermic peaks, with the peak for S2 being less distinct. It is speculated that this is also attributed to the decomposition of Ag_2_O, indicating that the surface content of Ag_2_O follows the order S1 > S3 > S2. The highest Ag_2_O content on the surface of silver powder S1 may be related to its higher surface energy, while the lower Ag_2_O content on the surface of S2 may be associated with the presence of residual organic compounds that hinder the oxidation of silver at room temperature. The pre-melting sintering reaction of silver powder is another exothermic process influenced significantly by the surface energy of the powder. In Figure 4, the DSC curve for silver powder S1 exhibits an increased slope around 450 °C, suggesting the initiation of sintering. Similarly, the sintering onset temperature for silver powder S2 is observed at approximately 500 °C, while that for silver powder S3 falls within the range of 550–600 °C.

2Ag_2_O → 2Ag + O_2_↑ (1)

### 3.2. Effect of Different Silver Powders on Sintering Properties of Silver Slurry

#### 3.2.1. Analysis of Square Resistance and Adhesion of Silver Paste after Sintering

**Figure 5:**
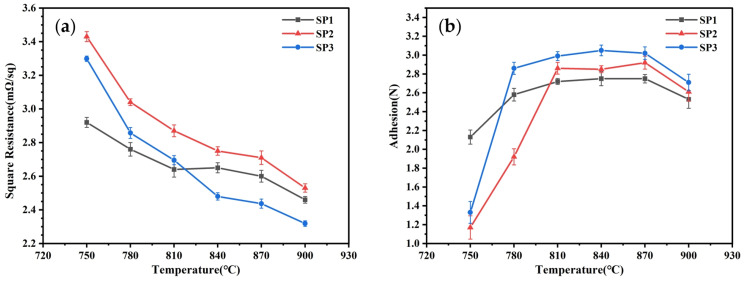
Properties of three silver pastes sintered at different peak temperatures, (**a**) square resistance, (**b**) adhesion.

Silver powders S1–S3 were formulated into silver pastes (SP1–SP3) with a mass ratio of silver powder to glass powder to organic carrier of 43:1:6. The pastes were then sintered at various peak temperatures to investigate the impact of silver powder sintering performance on the sheet resistance and adhesion of the resulting silver electrodes. The measurement results for sheet resistance and adhesion are presented in Figure 5. At lower sintering temperatures of 750 °C, SP1 exhibited superior sintering performance compared to SP2 and SP3. SP1 demonstrated lower sheet resistance of 2.92 mΩ/sq and higher adhesion of 2.13 N, indicating better sintering activity for the SP1 silver powder at lower temperatures, consistent with the results from DSC. As the sintering temperature gradually increases, the sheet resistance of SP2 and SP3 rapidly decreases, accompanied by a rapid increase in adhesion. At 810 °C, the sheet resistance of SP3 approached that of SP1, measuring 2.76 mΩ/sq and 2.64 mΩ/sq, respectively. By raising the temperature to 840 °C, the sheet resistance of SP3 was lower than that of SP1, measuring 2.56 mΩ/sq and 2.65 mΩ/sq, respectively. The sheet resistance of SP2 consistently remained higher than that of SP1, and only after sintering at 840 °C did it approach that of SP1. Additionally, at 810 °C, the adhesion of SP2 and SP3 surpassed that of SP1, measuring 2.86 N, 2.99 N and 2.72 N, respectively. After sintering at 810 °C, the variation in adhesion among the three silver pastes becomes minor, with a noticeable decline only at 900 °C. Considering the sheet resistance and adhesion of the three silver pastes at different sintering temperatures, it is evident that the sintering performance of SP1 surpasses that of SP2 and SP3 when the sintering temperature is below 810 °C. However, when the sintering temperature exceeds 810 °C, the sintering performance of SP3 becomes superior to that of SP1 and SP2.

#### 3.2.2. Surface and Section Micromorphology of Silver Paste after Sintering

**Figure 6:**
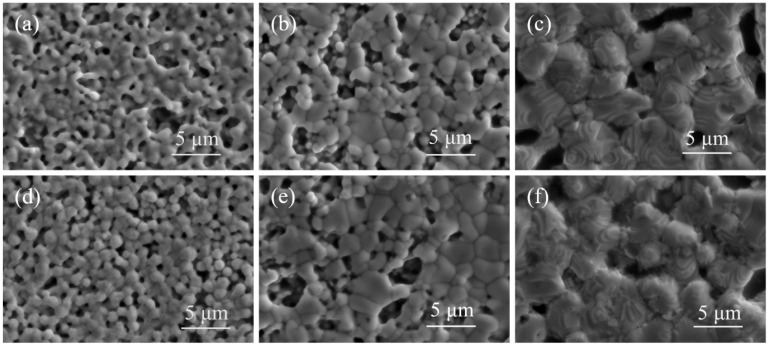
SEM images of silver paste Sp1 and SP3 sintered at different peak temperatures, (**a**) 750 °C, SP1; (**b**) 840 °C, SP1; (**c**) 900 °C, SP1; (**d**) 750 °C, SP3; (**e**) 840 °C, SP3; (**f**) 900 °C, SP3.

**Figure 7:**
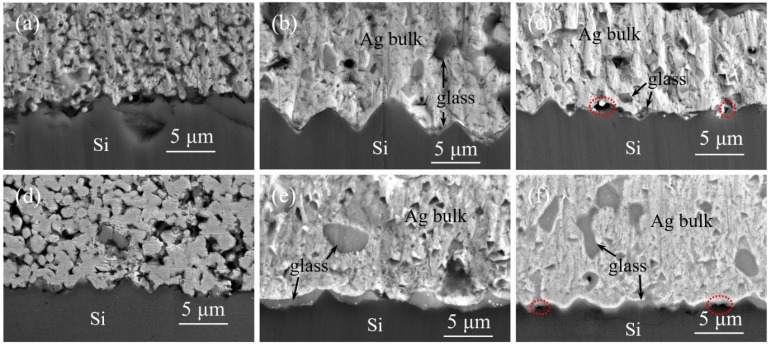
SEM cross sections of silver pastes Sp1 and SP3 sintered at different peak temperatures, (**a**) 750 °C, SP1; (**b**) 840 °C, SP1; (**c**) 900 °C, SP1; (**d**) 750 °C, SP3; (**e**) 840 °C, SP3; (**f**) 900 °C, SP3.

Due to the suboptimal sintering performance of silver paste SP2 at both low and high-temperature ranges, distinctive silver pastes SP1 and SP3 were selected for observation via SEM, focusing on their surface and cross-sectional microstructures after sintering at 750 °C, 840 °C, and 900 °C, as depicted in Figure 6 and Figure 7, respectively. Comparing Figure 6a,d, at 750 °C sintering, the necking between silver powder S1 particles has largely disappeared, indicating a trend toward mutual fusion and the formation of a conductive network, resulting in lower sheet resistance. In contrast, silver powder S3 exhibits partially formed necks between particles, with some particles just making contact without starting the sintering process, leading to a higher sheet resistance. Observing Figure 6b,e at 840 °C sintering, both silver powders S1 and S3 have entered the sintering shrinkage stage, with silver particles starting to fuse and grow. Due to the higher tap density of silver powder S3, resulting in a denser initial packing, the sintered layer of S3 demonstrates superior density and lower sheet resistance. Examining Figure 6c,f at 900 °C sintering, a melting tendency is observed in the silver powders, with crystal growth steps rapidly forming on the particle surfaces. The initial packing density of the silver powder begins to dominate over the sintering activity, leading to a modest decrease in sheet resistance for all three silver pastes, with SP3 exhibiting the lowest sheet resistance.

Comparing Figure 7a,d, at 750 °C sintering, the cross-sectional observation of the silver powder aligns with the surface findings. In the case of silver powder S3, the particles are not fully interconnected, resulting in fractures within the silver layer upon detachment, indicating poor adhesion for silver paste SP3. Contrasting Figure 7b,e at 840 °C sintering, the silver powder has achieved relatively dense sintering, with channels between the particles for glass powder flow closed. Some glass melt remains trapped within the silver layer, particularly noticeable in silver paste SP1 due to its higher sintering activity, resulting in a thicker glass layer between the silver layer and silicon. Consequently, SP1 exhibits higher sheet resistance and lower adhesion compared to SP3. Examining Figure 7c,f at 900 °C sintering, the closure of the channels in the silver powder accelerates, increasing the glass content within the silver layer and reducing the glass layer between the silver layer and the silicon. This leads to a decrease in adhesion for all three silver pastes. Additionally, at higher temperatures, the reaction between the glass and the SiN_x_ anti-reflective layer becomes more intense, generating gas due to the reaction. As the channels within the silver layer close prematurely, some gas remains trapped within the glass layer, forming voids, as outlined by the red dashed circles in the diagram. This phenomenon further contributes to the reduction in adhesion, potentially leading to delamination beneath the silver layer.

### 3.3. Effects of Different Silver Powders on the Morphology and Electrical Properties of Solar Cells

**Figure 8:**
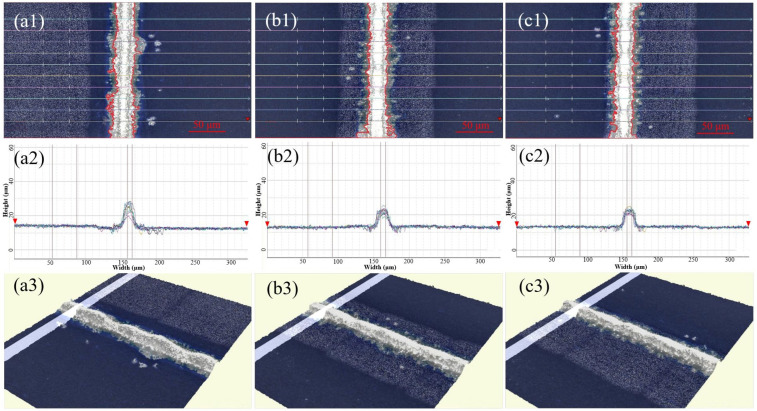
Three-dimensional microscopic surface images of silver grids of solar cells using different silver pastes, (**a1**) SP1; (**b1**) SP2; (**c1**) SP3l; overlap images of cross section of silver grids of solar cells used different silver pastes, (**a2**) SP1; (**b2**) SP2; (**c2**) SP3; 3D images of silver grids of solar cells used different silver pastes, (**a3**) SP1; (**b3**) SP2; (**c3**) SP3.

Silver pastes, SP1–SP3, were printed onto solar cells using a mesh screen with a fine grid width of 15 μm. After sintering at 840 °C, the morphology of the grid lines was examined using a 3D digital microscope, and the aspect ratio was measured, as depicted in Figure 8 and summarized in Table 3. Figure 8 reveals distinct segments in the silver grid lines of SP1, whereas those of SP2 and SP3 appear more uniformly flat. The cross-sectional overlap images in Figure 8(a2–c2) indicate good alignment of the contour lines for SP2 and SP3. Although SP1 exhibits significant variations in peak heights, even the smallest contour lines cover a sufficient area for effective electrical conduction, preventing grid line breaks. Given that silver powders S1–S3 undergo the same surface treatment process and exhibit similar shapes and particle sizes when processed into silver pastes (SP1–SP3), their printing characteristics are comparable. However, due to differences in the compactness of particle stacking after drying, SP1 displays the lowest stacking density. Additionally, owing to the high surface energy and sintering activity of silver powder S1, SP1 experiences the most pronounced sintering shrinkage, resulting in substantial axial contraction stress in the grid lines. Beyond a certain limit, this stress leads to segmented contraction of the grid lines.

The data for the gate lines height and width in Table 3 were obtained from the contour lines in Figure 8(a2–c2). The height of the gate line is determined by the difference between the height value of the top of the gate line and the baseline marked with a red inverted triangle. The width of the gate line is calculated as the difference between the values on both sides of the gate lines. Table 3 reveals that the aspect ratios of the silver grid lines in SP1–SP3 are comparable and generally high. This characteristic is advantageous for minimizing both the shading area and electrical resistance of the silver grid lines. Consequently, it contributes to the reduction in series resistance in solar cells, thereby enhancing the photoelectric conversion efficiency. Notably, SP3 exhibits the highest aspect ratio among the sintered silver pastes, while SP2 demonstrates the most uniform size distribution of silver grid lines. The narrowest width of the sintered grid lines among the three pastes is 4.49 μm, with a corresponding height of 17.8 μm, confirming their satisfactory conductivity.

The I–V performance tests were conducted on the solar cells printed with silver pastes SP1–SP3, and the results are presented in Table 4. It is evident that the three silver pastes exhibit minimal influence on the parallel resistance (R_sh_) and open-circuit voltage (V_oc_) of the solar cells. SP1 shows the lowest series resistance (R_s_) and the highest short-circuit current (I_sc_). This outcome may be attributed to the high surface energy of silver powder S1, leading to a higher proportion of silver entering the glass melt during sintering. Consequently, a large number of silver nanocrystals precipitate in the glass layer between the silver layer and silicon, resulting in low contact resistance, lower Rs, and higher Isc in the solar cells. SP3 demonstrates the highest fill factor (FF), possibly due to its silver grid lines having the maximum aspect ratio, minimizing shading and, thus, increasing FF. In terms of overall photoelectric conversion efficiency, both SP1 and SP3 are comparable and outperform SP2.

## 4. Conclusions

In situations where the shape and size of silver powders are similar, the compositional structure significantly influences their performance. The crystal growth silver powder exhibited inferior sintering activity, but had the highest tap density of 5.52 g/cm^3^. The resulting silver paste demonstrated excellent densification during sintering, with optimal performance achieved at 840 °C, yielding a sheet resistance of 2.56 mΩ/sq and adhesion of 3.05 N, surpassing the other silver pastes. The uniform and high aspect ratio (4.0) of the sintered solar cell electrode grid lines contributed to a reduced shading area and a high fill factor of 81.59%, resulting in a slightly elevated photoelectric conversion efficiency of 23.17% compared to the polycrystalline aggregated silver powder. The crystal growth silver powder exhibited heightened sintering activity at temperatures of 840 °C and above. Due to its high particle-packing density and minimal sintering shrinkage, its incorporation into silver paste enables the production of electrode grid lines with greater aspect ratios and reduced shading areas, conducive to enhanced photoelectric conversion efficiency. Therefore, when formulating silver paste with higher sintering temperatures, preference can be given to the crystal growth silver powder. Polycrystalline aggregated silver powder demonstrates superior sintering activity at lower temperatures, with a sintering initiation temperature of around 450 °C. The resulting silver paste, sintered at 750 °C, exhibits low sheet resistance of 2.92 mΩ/sq and high adhesion of 2.13 N. Although the solar cell electrode grid lines possess a high aspect ratio of 0.37, they have poorer uniformity. The high sintering activity of the silver powder leads to the dissolution of the glass layer and increased silver deposition. Consequently, the paste exhibits excellent conductivity, low contact resistance of the silver electrode of 1.23 mΩ, high series resistance of the solar cell of 23.16%, and a photoelectric conversion efficiency of 23.16%. Polycrystalline aggregated silver powder, when combined with glass powder with a low melting temperature and good melt flow, can be employed to formulate silver pastes with sintering windows around 750 °C. This effectively mitigates the impact of high-temperature processing on the diffusion concentration of the solar cell emitter, thereby enhancing the photoelectric conversion efficiency. Thus, when preparing silver paste with lower sintering temperatures, preference can be given to polycrystalline aggregated silver powder. The study in this paper provides valuable insights for selecting silver powders for formulating solar cell silver pastes with different sintering windows.

## Figures and Tables

**Table 1:** Diffraction peak intensity and FWHM of different crystal planes of silver powder.

Sample	(111) Facet		(200) Facet		(220) Facet	
	Intensity	FWHM	Intensity	FWHM	Intensity	FWHM
S1	2203	0.343	535	0.505	427	0.412
S2	2608	0.300	709	0.480	448	0.506
S3	3454	0.298	998	0.450	654	0.452

**Table 2:** Macroscopic physical properties of silver powder S1–S3.

Sample	Average Particle Size (μm)	Tap Density (g/cm^3^)	Specific Surface Area (m^2^/g)	Ignition Loss (%)
S1	1.23	3.34	0.92	0.87
S2	1.26	4.67	0.67	0.63
S3	1.06	5.52	0.41	0.35

**Table 3:** The height, width and aspect ratio of silver grids of solar cells using different silver pastes.

Cross Section	SP1			SP2			SP3		
	Height (μm)	Width (μm)	H/W Ratio	Height (μm)	Width (μm)	H/W Ratio	Height (μm)	Width (μm)	H/W Ratio
1	10.07	27.86	0.36	9.68	25.80	0.38	7.89	22.20	0.36
2	12.23	40.51	0.30	8.02	23.20	0.35	9.88	22.20	0.45
3	12.80	36.40	0.35	6.00	23.74	0.25	9.21	25.54	0.36
4	4.49	17.80	0.25	10.37	23.48	0.44	6.90	25.00	0.28
5	10.30	22.40	0.46	7.99	24.51	0.33	11.45	23.20	0.49
6	10.10	25.54	0.40	11.82	26.60	0.44	7.44	29.93	0.25
7	10.94	33.80	0.32	9.57	26.32	0.36	9.69	18.10	0.54
8	13.54	29.20	0.46	7.48	21.16	0.35	9.06	22.40	0.40
9	7.21	19.90	0.36	8.68	21.93	0.40	7.59	20.60	0.37
10	8.66	22.70	0.38	8.37	23.20	0.36	10.03	21.16	0.47
Min	4.49	17.80	0.25	6.00	21.16	0.25	6.90	18.10	0.25
Max	13.54	40.51	0.46	11.82	26.60	0.44	11.45	29.93	0.54
Mean	10.03	27.61	0.37	8.80	24.00	0.37	8.92	23.04	0.40

**Table 4:** I–V performance test results of solar cells using different silver pastes.

Paste	I_sc_ (A)	V_oc_ (V)	R_s_ (mΩ)	R_sh_ (Ω)	FF (%)	Eta (%)
SP1	13.67	0.6869	1.23	847	81.37	23.15
SP2	13.62	0.6872	1.57	848	81.42	23.09
SP3	13.64	0.6870	1.43	853	81.59	23.17

## Data Availability

Data are contained within the article.

## Supplementary Materials

The following supporting information can be downloaded at: https://www.mdpi.com/article/10.3390/ma17020445/s1, Figure S1: DSC-TG curve of silver powders, (a) S1, (b) S2, (c) S3.
