# Supplementary material for: Effect of Silver Powder Microstructure on the Performance of Silver Powder and Front-Side Solar Silver Paste
Source: Materials (Basel). 2024 Jan 17;17(2):445. doi: 10.3390/ma17020445 (PMC10820057; doi:10.3390/ma17020445)
Supplement: Supplementary file 1 [file materials-17-00445-s001.zip › materials-2816572-supplementary.pdf]

The DSC-TG test results for silver powders S1-S3 are illustrated in Figure S1. In Figure 4 of the manuscript, the DSC curves of silver powders S1-S3 were placed in one figure, so the TG curves were omitted.

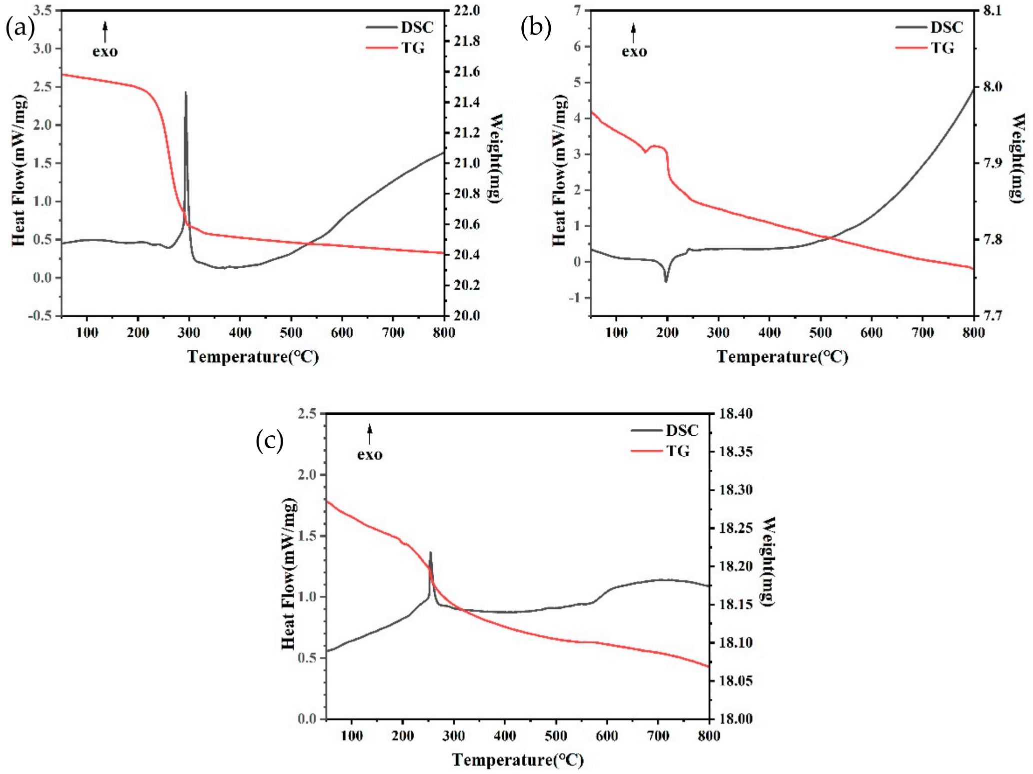

Figure S1. DSC-TG curve of silver powders, (a) S1, (b) S2, (c) S3.
